# Bladder Cancer detection by urinary methylation markers GHSR/MAL: a validation study

**DOI:** 10.1007/s00345-024-05287-5

**Published:** 2024-10-16

**Authors:** I. J. Beijert, Y. van den Burgt, A. E. Hentschel, J. Bosschieter, P. C. Kauer, B. I. Lissenberg-Witte, R. J.A. van Moorselaar, J. A. Nieuwenhuijzen, R. D.M. Steenbergen

**Affiliations:** 1 Department of Pathology, Cancer Center Amsterdam, Amsterdam UMC, Vrije Universiteit Amsterdam, Amsterdam, The Netherlands; 2 Department of Urology, Cancer Center Amsterdam, Amsterdam UMC, Vrije Universiteit Amsterdam, Amsterdam, The Netherlands; 3 Cancer Center Amsterdam, Imaging and Biomarkers, Amsterdam, The Netherlands; 4 Department of Urology, OLVG, Amsterdam, The Netherlands; 5 Department of Epidemiology and Data Science, Amsterdam UMC, location Vrije Universiteit Amsterdam, Amsterdam, The Netherlands

**Keywords:** (MeSH): bladder Cancer, DNA methylation, Liquid biopsy, Urine, Detection, Biomarker, Validation

## Abstract

**Purpose:**

Although cystoscopy is a reliable tool for detecting bladder cancer, it poses a high burden on patients and entails high costs. This highlights the need for non-invasive and cost-effective alternatives. This study aimed to validate a previously developed urinary methylation marker panel containing *GHSR* and *MAL*.

**Methods:**

We enrolled 134 patients who underwent cystoscopy because of hematuria, including 63 individuals with primary bladder cancer and 71 with non-malignant findings. Urine samples were self-collected at home and sent via regular mail. Subsequently, DNA was extracted and the hypermethylation of *GHSR* and *MAL* was evaluated using quantitative methylation-specific polymerase chain reaction. The performance of methylation markers was assessed using area-under-the-curve (AUC) analysis and sensitivity and specificity based on pre-established cut-off values.

**Results:**

Validation of the marker panel *GHSR/MAL* resulted in an AUC of 0.87 at 79% sensitivity and 80% specificity. Sensitivity was comparable to the previous investigation (*P* > 0.9), though specificity was significantly lower (*P* = 0.026). Sensitivity was higher for high-grade tumors compared to low-grade tumors (94% vs. 60%, *P* = 0.002).

**Conclusion:**

Validation of the *GHSR/MAL* methylation marker panel on at home collected urine samples confirms its robust performance for bladder cancer detection in a hematuria population, and underscores the diagnostic potential for future clinical application.

## Introduction

Bladder cancer (BC) is one of the most common urological malignancies [1]. Patients often present with painless hematuria, prompting the need for cystoscopy and transurethral resection of the bladder tumor (TURBT) to confirm diagnosis. Bladder cancer is categorized as either non-muscle-invasive BC (NMIBC) or muscle-invasive BC (MIBC). Patients with NMIBC require frequent surveillance as the disease often recurs (31–78%) or progresses (1–45%) [2]. While cystoscopy serves as a reliable tool for both initial diagnosis and surveillance, its invasiveness, costs, and risk of complications necessitates the exploration of cost-effective, non-invasive alternatives for the detection of BC to shift hospital care to primary care and improve patient well-being.

A urinary biomarker holds the promise of addressing this need. Despite numerous biomarkers being investigated and some being mentioned by international guidelines, the guidelines also mention that none of these markers have been accepted as routine practice due to insufficient diagnostic potential especially in low-grade (LG) disease. Achieving a balance between sensitivity and specificity across the spectrum of tumor stages and grades remains challenging [2, 3]. Additionally, most studied biomarkers are expensive, require immediate cold storage of urine, or are still awaiting validation in independent cohorts. The latter may potentially lead to misguided assumptions regarding their diagnostic promise. Therefore, further research is necessary to validate urinary biomarkers with robust diagnostic capabilities and easy applicability in clinical and outpatient settings, preferably through home sampling.

Identifying altered DNA methylation has emerged as a promising urinary biomarker source, given its pivotal early role in bladder cancer development by silencing tumor suppressor genes through hypermethylation [4–6]. In our prior preclinical study addressing technical aspects and involving 100 bladder cancer patients and 108 controls with and without hematuria, we identified hypermethylation of *GHSR/MAL* as urinary biomarkers with good diagnostic potential. The methylation marker panel demonstrated an AUC of 0.89 with 80% sensitivity and 93% specificity [7]. The aim of the current study is to validate the diagnostic accuracy of the *GHSR/MAL* marker panel for bladder cancer detection in a hematuria cohort, as this reflects its intended use in clinical practice.

## Materials and methods

### Patients

The inclusion criteria encompassed patients referred for microscopic or macroscopic hematuria. All patients underwent cystoscopy. Suspicion of primary bladder cancer was confirmed by subsequent transurethral resection of bladder tumor (TURBT). Patients with histologically confirmed urothelial carcinoma were classified as case. The control group consisted of patients with microscopic or macroscopic hematuria but no signs of bladder cancer (or upper tract urothelial cancer [UTUC]) at cystoscopy, abdominal ultrasound or CT scan, which are the recommended imaging modalities for hematuria patients according to the EAU Guidelines [2]. Patients with a prior history of malignancy, including previous bladder cancer, were excluded. Patients were included between November 2021 and March 2023 at Amsterdam University Medical Centers and OLVG.

The study protocol was approved by the Medical Ethics Committee (2018.355 [16-10-2018], WO 18.155 [21-12-2018]). All participants gave informed written consent for study participation prior to inclusion.

### Urine samples

Urine samples were collected by patients at home in tubes prefilled with ethylenediaminetetraacetic acid (EDTA) in a final concentration of 40 mM to maintain DNA quality [8]. In the patients scheduled for TURBT, samples were collected (at home) before surgery. Urine samples were sent by regular mail to the hospital and processed within 24–72 h. Urine pellet was obtained by centrifugation of 15 mL urine at 800xg for 10 min and subsequently stored at -20 °C until DNA isolation.

### DNA isolation, bisulfite conversion and quantitative methylation-specific PCR

DNA isolation of urine pellet was achieved using QIAamp DNA Mini Kit (Qiagen GmbH, Hilden, Germany) in accordance with manufacturers protocol. DNA concentrations were measured with NanoDrop 1000 (ThermoFisher Scientific, Waltham, MA, USA). Bisulfite conversion was conducted with EZ DNA Methylation Kit (Zymo Research, Orange, CA, USA). Quantitative methylation-specific PCR (qMSP) was performed for the target genes (*GHSR* and *MAL*) in a Epitect Multiplex PCR Mastermix (Qiagen, Venlo, Netherlands) as described previously [7, 9]. The sequences for the primers and probes have been previously described [7, 9]. Double-stranded gBlocks™ Gene Fragments (Integrated DNA Technologies) containing the target regions were used as a positive control and H2O was used as a negative control. The Ct values of the target genes were calculated by the comparative Ct (2^−ΔCT^ x100) method using ACTB as a reference gene. Results were considered invalid if ACTB Ct > 32 [10]. 

### Statistical analysis

Categorical data are presented as frequencies and percentages, and continuous data are presented as medians and interquartile range (IQR). The Chi-square test was used to compare categorical variables between cases and controls, and the Mann-Whitney U test was performed to compare continuous variables.

Methylation levels of the urinary markers were calculated as log2-transformed Ct ratios. The Mann-Whitney U test was used to compare methylation levels between the validation study and the previous preclinical study (cases and controls separately). The thresholds for positive or negative results for each marker were established in the preceding study [7]. The diagnostic performance (sensitivity/specificity) of the marker panel *GHSR/MAL* was established using a believe-the-positive-principle, similar to the prior study. The area-under-the-curve (AUC) was constructed using the slopes and intercept from the previous preclinical study. The AUCs of both studies were compared using a Delong test. Sensitivity and specificity of both studies were compared using a Chi-square test. Subgroup analyses for stage (NMIBC vs. MIBC), grade (LG vs. high-grade [HG]), multiplicity (solitary vs. multiple), size (< 3 cm vs. ≥ 3 cm) and gender (male vs. female) were conducted, and sensitivity of the groups were compared using the Chi-square test.

## Results

In total, 134 patients with hematuria were included, of whom 63 patients were diagnosed with primary bladder cancer, leaving 71 controls with hematuria. Patient characteristics are displayed in Table 1. Methylation results for *GHSR* from 8 controls and methylation results for *MAL* from 1 control were omitted from the analysis due to *ACTB* Ct values > 32.

**Table 1:** Patient and tumor characteristics

Characteristics	Control (*n* = 71)	Bladder Cancer (*n* = 63)	*P*-value
**Age in years** ***(IQR)***	65 (58–76)	72 (64–75)	0.2
**Gender**, *n (%)*			0.6
Male	51 (72)	49 (78)	
Female	20 (28)	14 (22)	
**Grade WHO 1973**, ***n (%)***			
G1		11 (18)	
G2		21 (34)	
G3		29 (48)	
Unknown		2	
**Grade WHO 2004/2016/2022**, ***n (%)***			
LG		25 (41)	
HG		36 (59)	
Unknown		2	
**Stage**, ***n (%)***			
Ta		37 (59)	
T1		12 (19)	
Tis		0 (0)	
≥ T2		13 (22)	
**Multiplicity**, ***n (%)***			
Single		40 (65)	
Multiple		22 (35)	
Unknown		1	
**Tumor size**, ***n (%)***			
< 3 cm		40 (69)	
≥ 3 cm		18 (31)	
Unknown		5	
**Concomitant CIS**, ***n (%)***			
Yes		5 (8)	
No		57 (92)	

Abbreviations IQR: Interquartile range; G: grade; HG: high-grade; LG low-grade; WHO: World Health organization; CIS: carcinoma in situ

### DNA methylation levels

**Fig. 1:**
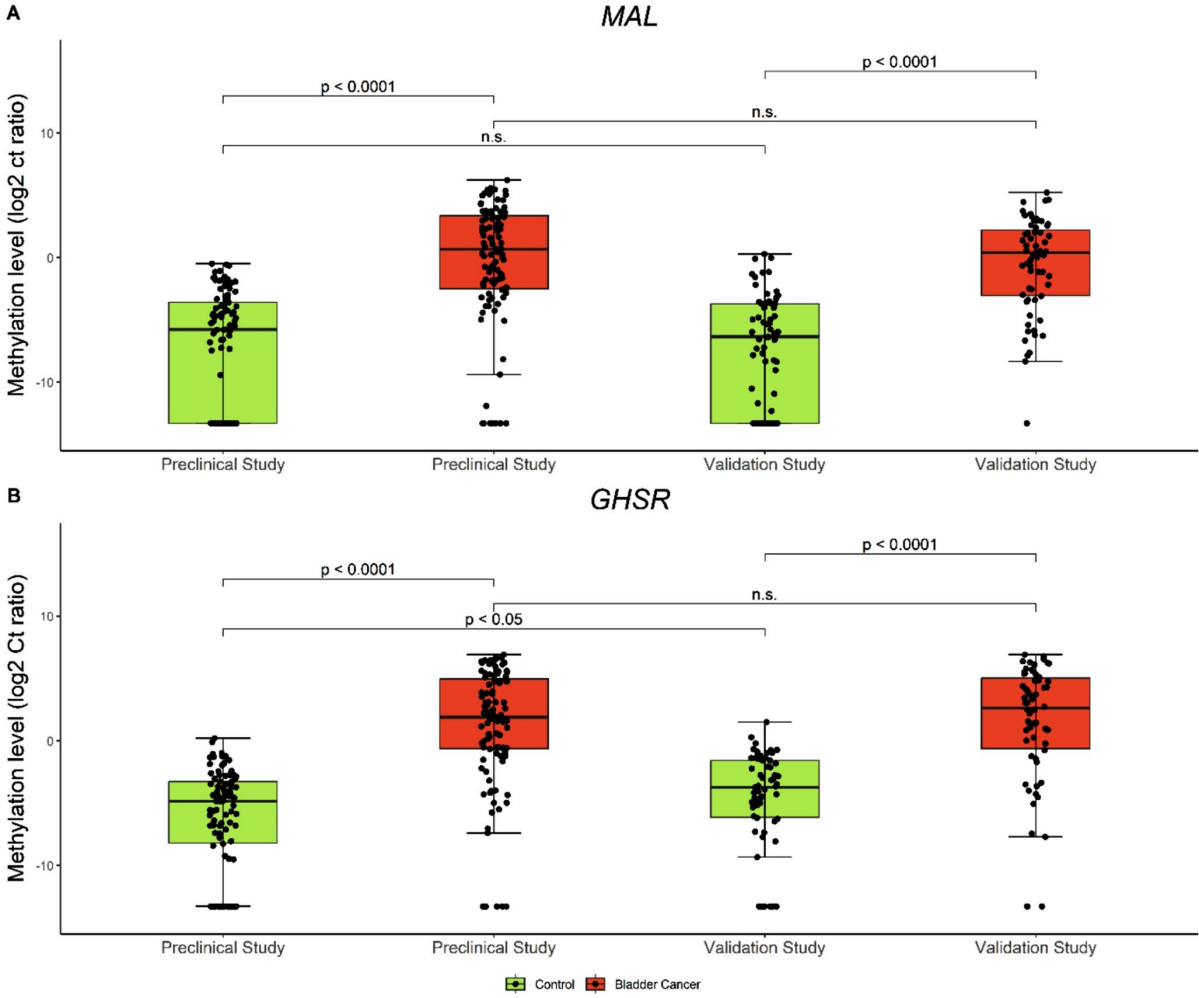
Methylation levels of *GHSR/MAL* in the preclinical versus the validation study. Methylation levels of *GHSR* (**A**) and *MAL* (**B**) were significantly higher in cases compared to controls. Methylation levels of *GHSR* and *MAL* in bladder cancer patients were not significantly different in the validation cohort compared to the preclinical study (*P* = 0.6 and *P* = 0.3, respectively). In controls, methylation levels of *MAL* were not significantly different in the validation cohort (*P =* 0.3*)*, though methylation levels for *GHSR* were significantly higher (*P =* 0.015)

The methylation levels of *GHSR* and *MAL* exhibited a marked increase in urine of bladder cancer patients compared with hematuria controls (*P* < 0.001 for both *GHSR* and *MAL*) (Fig. 1). The methylation levels of *GHSR* and *MAL* in bladder cancer patients in this validation study were consistent with the earlier findings from the previous preclinical study, showing no significant differences (*P* = 0.6 and *P* = 0.3, respectively). Furthermore, no significant differences were observed in the methylation levels of *MAL* among controls (*P* = 0.3), whereas *GHSR* methylation levels showed an increase in controls in this validation study (*P* = 0.015) (Fig. 1).

### Diagnostic performance of *GHSR/MAL* methylation analysis for the detection of bladder cancer

**Fig. 2:**
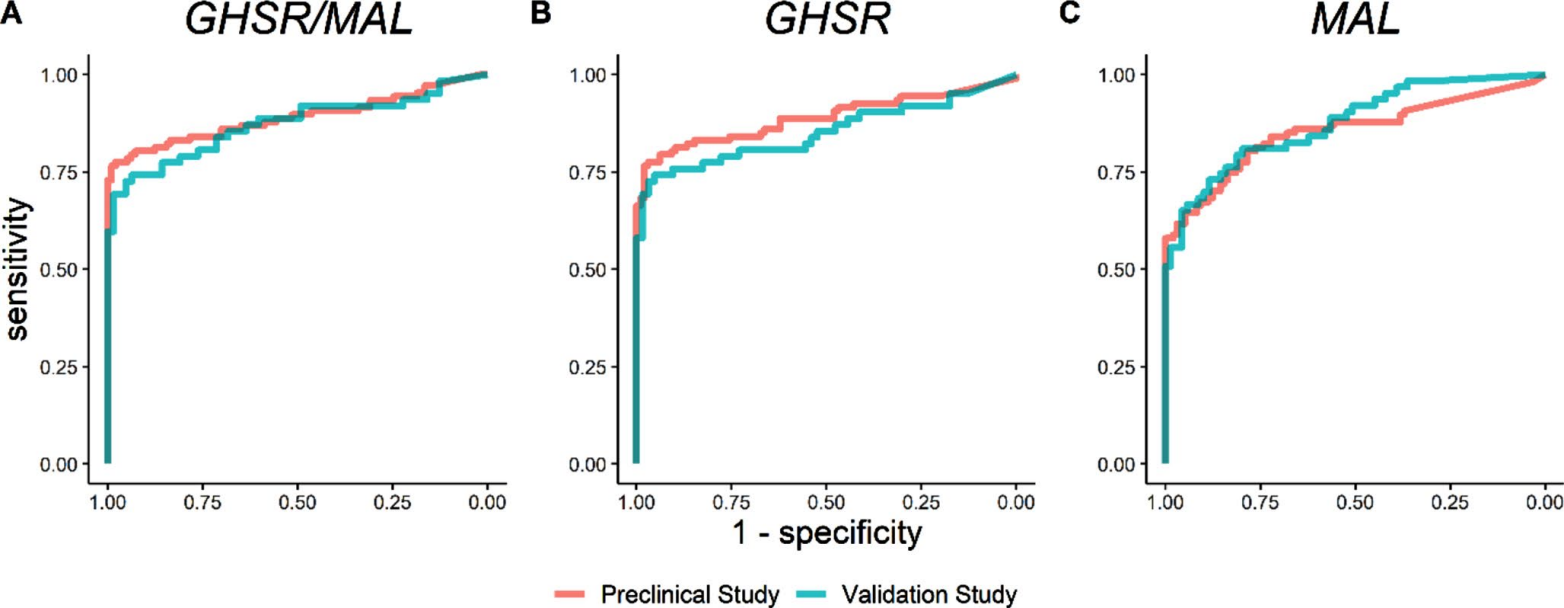
ROC of individual markers *GHSR* and *MAL* and marker panel *GHSR/MAL* in the preclinical study and validation study. There was no significant difference in AUC for marker panel (*GHSR/MAL P* = 0.7) or the individual markers (*GHSR P* = 0.4, *MAL P* = 0.6*)* between both studies. *Abbreviations: ROC: Receiver Operating Curve; AUC: Area Under the Curve*

The marker panel *GHSR/MAL* yielded an AUC of 0.87 (95%CI 0.81–0.94), consistent with the previous preclinical study (*P* = 0.7). (Fig. 2) The corresponding sensitivity and specificity of the *GHSR/MAL* marker panel were determined using previously established cut-off values and the believe-the-positive principle. Sensitivity showed no significant difference (79% vs. 80%, *P* > 0.9), while specificity was significantly lower (80% vs. 93%, *P* = 0.026) compared to the previous preclinical study (Table 2).

For individual markers, an AUC of 0.85 (95%CI 0.78–0.93) was observed for *GHSR*, which was comparable to the preclinical study (*P* = 0.4). For *MAL*, an AUC of 0.88 (95%CI 0.82–0.93) was found, mirroring the preclinical study as well (*P* = 0.6). (Fig. 2) Corresponding sensitivities showed no significant differences for both *GHSR* (76% vs. 77%, *P* > 0.9) and *MAL* (66% vs. 64%, *P* = 0.5). Specificity showed no significant difference for *MAL* (91% vs. 95%, *P* = 0.5), although it was significantly lower for *GHSR* (87% vs. 98%, *P* = 0.014). (Table 2)

**Table 2:** Diagnostic performance of the DNA methylation markers

	Preclinical Study (7)	Validation Study	*P*-value
GHSR			
AUC	0.89 (95% CI 0.84–0.94)	0.85 (95% CI 0.78–0.93)	0.4
Sensitivity	0.77 (95% CI 0.67–0.84)	0.76 (95% CI 0.65–0.85)	> 0.9
Specificity	0.98 (95% CI 0.95-1.00)	0.87 (95% CI 0.79–0.95)	0.014
***MAL***			
AUC	0.85 (95% CI 0.80–0.91)	0.88 (95% CI 0.82–0.93)	0.6
Sensitivity	0.64 (95% CI 0.55–0.73)	0.66 (95% CI 0.54–0.77)	0.9
Specificity	0.95 (95% CI 0.90–0.99)	0.91 (95% CI 0.84–0.97)	0.5
***GHSR/MAL***			
AUC	0.89 (95% CI 0.84–0.94)	0.87 (95% CI 0.81–0.94)	0.7
Sensitivity	0.80 (95% CI 0.71–0.87)	0.79 (95% CI 0.68–0.89)	> 0.9
Specificity	0.93 (95% CI 0.85–0.97)	0.80 (95% CI 0.69–0.89)	0.026

Abbreviations AUC: Area Under the Curve; 95% CI: 95% Confidence Interval

#### Diagnostic performance in relation with tumor stage, grade, multiplicity, size and gender

The diagnostic performance in relation to various clinical parameters, such as tumor stage, tumor grade, tumor multiplicity, size, and gender, was assessed. Sensitivity of *GHSR/MAL* in LG tumors was 60%, which was significantly lower than in HG tumors (94%, *P* = 0.003*)*. Sensitivity for *GHSR/MAL* in HG/G3 tumors was 100%. No significant differences were found in other subgroups. (Table 3)

**Table 3:** Sensitivity in subgroups

	GHSR	MAL	GHSR/MAL
**Stage**			
*NMIBC*	0.70 (95% CI 0.58–0.83)	0.65 (95% CI 0.51–0.78)	0.76 (95% CI 0.63–0.88)
*MIBC*	0.93 (95% CI 0.79-1.00)	0.71 (95% CI 0.50–0.93)	0.93 (95% CI 0.79-1.00)
**Grade**			
*LG*	0.5 (95% CI 0.29–0.71)*	0.44 (95% CI 0.24–0.64)*	0.60 (95% CI 0.40–0.80)*
*HG*	0.94 (95% CI 0.86-1.00)*	0.83 (95% CI 0.69–0.94)*	0.94 (95% CI 0.86-1.00)*
**Multiple**			
*Single*	0.72 (95% CI 0.56–0.85)	0.63 (95% CI 0.48–0.78)	0.75 (95% CI 0.60–0.88)
*Multiple*	0.82 (95% CI 0.63–0.95)	0.73 (95% CI 0.55–0.91)	0.86 (95% CI 0.68-1.00)
**Size**			
*<3 cm*	0.67 (95% CI 0.51–0.79)	0.55 (95% CI 0.40–0.70)	0.73 (95% CI 0.58–0.85)
*≥3 cm*	0.89 (95% CI 0.72-1.00)	0.83 (95% CI 0.67-1.00)	0.89 (95% CI 0.72-1.00)
**Gender**			
*Male*	0.77 (95% CI 0.65–0.88)	0.73 (95% CI 0.61–0.84)	0.80 (95% CI 0.67–0.90)
*Female*	0.71 (95% CI 0.43–0.93)	0.43 (95% CI 0.14–0.71)	0.79 (95% CI 0.57-1.00)

Abbreviations NMIBC: Non-Muscle-invasive Bladder Cancer; MIBC: Muscle-invasive Bladder Cancer; LG: low-grade according to World Health Organization 2004/2016/2022 grading system; HG: high-grade according to World Health Organization 2004/2016/2022 grading system; 95% CI: 95% Confidence Interval

*P < 0.05

## Discussion

An accurate home-based urinary assay holds the promise to change the diagnostic pathway of bladder cancer detection by (partially) replacing cystoscopy and shifting care from hospitals to primary care. This validation study confirmed the diagnostic performance of the methylation marker panel *GHSR/MAL* for detecting bladder cancer among patients with hematuria using urine samples collected at home. The study yielded a similar AUC of 0.87 (preclinical study: 0.89) and maintained a comparable sensitivity of 79% (preclinical study: 80%). However, it resulted in a lower specificity of 80% (preclinical study: 93%).

Validation studies frequently cannot replicate the initially reported diagnostic accuracy [4]. For instance, while Renard et al. initially demonstrated a sensitivity of 90% and 93% specificity for *TWIST1* and *NID2* for primary bladder tumors, subsequent multi-institutional validation revealed a sensitivity of 67% and specificity of 61% for primary and recurrent bladder tumors [11, 12]. Additionally, Bladder EpiCheck (encompassing 15 methylation markers) initially demonstrated a sensitivity of 90% for recurrent tumors, but subsequent validation studies report sensitivities ranging from 62 to 68% [13–15]. In our validation study, specificity notably reduced to 80% compared to the previous 93% due to higher methylation levels of the controls in the validation cohort. This disparity is likely attributed to the different control groups used: the preclinical study included both healthy individuals and those with hematuria, while this validation study exclusively comprised patients referred to urologists for hematuria. This could lead to an increase in false positives, where individuals with benign conditions are mistakenly identified as having cancer. This may be due to the benign condition itself or because the biomarker is detecting pre-malignant lesions that have not yet progressed to bladder cancer and are undetectable by cystoscopy or imaging. An alternative possibility is that patients presenting with hematuria may have clinically undetected UTUC, which could be identified by our biomarkers. This results in a reduced specificity by having more ‘false positives’ that are, in fact, true positives. Reduced specificity in bladder cancer biomarkers leads to unnecessary cystoscopies, which are invasive and costly. Nonetheless, without a biomarker all these patients would be scheduled for a cystoscopy. More important, sensitivity remained consistent with the preclinical study. Considering the exclusive focus on primary tumors in this validation study, whereas the previous preclinical study included approximately 30% of recurrent bladder cancer cases, which often exhibit a lower tumor burden, a higher sensitivity might have been expected. Yet, tumors in this validation study were smaller (69% vs. 55% were < 3 cm) and exhibited less CIS (5% vs. 11%) compared to those in the preclinical study, possibly leading to a reduced sensitivity. This underscores the significance of carefully assessing the variability in both tumor and patient characteristics within the study cohort and emphasizes the necessity for prospective validation in large, independent cohorts prior to implementation.

The previously observed (and unexplained) higher sensitivity in males compared to females in the preclinical study was not found in this validation study. Consistent with our previous study and several other biomarker studies [13–17], sensitivity increased with higher tumor grades (60% for LG, 94% for HG and 100% for HG/G3). This may reflect underlying genetic and epigenetic changes, including DNA hypermethylation, in higher grade tumors that do not occur in less aggressive LG tumors [18–20]. Additionally, since we solely focused on DNA hypermethylation, we might not capture the heterogeneity that characterizes the (epi)genomics of bladder cancer. Integrating a complementary biomarker with higher sensitivity for LG tumors into a combined assay could potentially enhance overall sensitivity.

Promising relatively novel and commercially available urinary biomarker tests for the primary detection of bladder cancer include for example *Xpert bladder Cancer (*mRNA’s: *CRH*,* IGF2*,* UPK1B*,* ANXA10*,* ABL1*) with a sensitivity of 73–78% at 77–84% specificity [21, 22], *ADXBLADDER* (MCM5 antibodies) at 60–73% sensitivity and 68–88% specificity [23, 24], *Uromonitor* (mutations in *TERT*,* FGFR3*,* KRAS*) with 50% sensitivity and 100% specificity [25], *Cx bladder* (mRNA’s: *MDA*,* HOCXA13*,* CDC2*,* IGFBP5* and *CXCR2*) with 82–96% sensitivity and 34–85% specificity [26, 27] and *AssureMdx* (mutations in *FGFR3*,* TERT*,* HRAS* combined with methylation of *OTX1*,* ONECUT2*,* TWIST1*) at 92–93% sensitivity and 73–86% specificity [16, 28]. Our *GHSR/MAL* methylation marker panel demonstrates diagnostic accuracy on par with these commercial tests. Nevertheless, the question arises: is a sensitivity of 79%, combined with 80% specificity, good enough to meet the criteria for clinical implementation? Necessities for sensitivity and specificity largely depend on the intended clinical application. For the detection of primary BC, prioritizing high sensitivity across all tumor stages and grades is crucial in order not to miss any cancers. Lower specificity may be acceptable, as without a biomarker, every patient would be scheduled for cystoscopy. However, in a follow-up approach that alternates between cystoscopy and biomarkers, sensitivity becomes particularly crucial for HG disease. HG tumors progress to muscle-invasive disease more rapidly and frequently, necessitating early intervention. Given that LG tumors are less likely to progress, a missed LG tumor will be detected at the next cystoscopy, without affecting prognosis. Apart from reliability, a biomarker assay needs to be practical for integration into standard diagnostic labs, economically viable, and considerate of patient viewpoints. Most bladder cancer patients are reluctant to switch from cystoscopy to a urinary test unless the test achieves at least 95% sensitivity, which poses a considerable challenge [29, 30]. Unfortunately, none of the currently available commercial tests can fulfill these criteria and entirely substitute cystoscopy. Even though the utilization of our markers in primary detection settings might be hindered by sensitivity of 60% for LG disease at the current set specificity, our assay does facilitate home-based testing and most likely cost-effective integration into routine diagnostic laboratories. Given the flexibility of cut-off values, we have the opportunity to boost sensitivity by decreasing specificity in a primary detection setting. Furthermore, our markers could offer added value in surveillance, with a 94% detection rate for HG and 100% for HG/G3 tumors, though further validation within a surveillance context is necessary.

The study’s limitations encompass its case enriched design, which diverges from routine clinical practice, thereby limiting the assessment of diagnostic accuracy to sensitivity and specificity without considering negative or positive predictive value, which rely on disease prevalence. Additionally, we have validated these DNA methylation markers for primary bladder cancer detection, so further prospective validation is warranted in patients undergoing bladder cancer surveillance. Recurrent tumors are often smaller in size and LG (as HG tumors are more frequently muscle-invasive requiring different treatment and follow-up). Smaller tumors likely release less tumor DNA and LG tumors exhibit fewer molecular abnormalities, potentially resulting in reduced sensitivity. A third limitation is that *GHSR* results from 8 controls were deemed invalid as the *ACTB* values were above 32. We hypothesize that in the absence of tumor and when urine is diluted, the concentration of urinary DNA becomes too low. Van Kessel et al. outlined a higher DNA concentration for tumor-positive urine samples compared to non-malignant samples [16] and in a subsequent study, the methylation and/or mutation status of all markers could not be determined in 17% of samples included [28]. This issue appears to be widespread, as demonstrated in the study by Pierconti et al., where 11–14% of samples tested with Bladder EpiCheck in a surveillance cohort were invalid [31]. Most of our invalid samples for *GHSR* were valid for *MAL* which could potentially be attributed to repeated thawing of bisulfite treated DNA. Key advantages of our assay are its affordable and straightforward-to-implement biomarkers, compatible with convenient at-home sampling and a shift from hospital to primary care.

## Conclusions

Validation of the *GHSR/MAL* methylation marker panel in a hematuria cohort confirms the findings of our previous preclinical study with robust test performance. This emphasizes the diagnostic potential of *GHSR/MAL* methylation testing in at-home collected urine for future clinical application.

## Data Availability

Data will be made available upon reasonable request.
